# Investigating the Effectiveness of Ceragenins against *Acinetobacter baumannii* to Develop New Antimicrobial and Anti-Adhesive Strategies

**DOI:** 10.3390/ijms25137036

**Published:** 2024-06-27

**Authors:** Maciej Karasiński, Urszula Wnorowska, Tamara Daniluk, Piotr Deptuła, Milena Łuckiewicz, Paulina Paprocka, Bonita Durnaś, Karol Skłodowski, Beata Sawczuk, Paul B. Savage, Ewelina Piktel, Robert Bucki

**Affiliations:** 1 Department of Medical Microbiology and Nanobiomedical Engineering, Medical University of Białystok, 15-222 Białystok, Poland; maciek.karasinski@gmail.com (M.K.); urszula.wnorowska@umb.edu.pl (U.W.); tamara.daniluk@umb.edu.pl (T.D.); karol.sklodowsky@gmail.com (K.S.); 2 Independent Laboratory of Nanomedicine, Medical University of Białystok, 15-222 Białystok, Poland; piotr.deptula@umb.edu.pl (P.D.); milenaluckiewicz@gmail.com (M.Ł.); ewelina.piktel@umb.edu.pl (E.P.); 3 Department of Microbiology and Immunology, Institute of Medical Science, Collegium Medicum, Jan Kochanowski University in Kielce, 25-317 Kielce, Poland; paulina.paprocka@ujk.edu.pl (P.P.); bonita.durnas@ujk.edu.pl (B.D.); 4 Department of Prosthodontics, Medical University of Bialystok, Sklodowskiej 24a, 15-276 Bialystok, Poland; beata.sawczuk@wp.pl; 5 Department of Chemistry and Biochemistry, Brigham Young University, Provo, UT 84602, USA; pbsavage@chem.byu.edu

**Keywords:** *Acinetobacter baumannii*, ceragenins, biofilm, atomic force microscopy, antimicrobial, anti-adhesive strategies

## Abstract

A growing body of experimental data indicates that ceragenins (CSAs), which mimic the physicochemical properties of the host’s cationic antimicrobial peptide, hold promise for the development of a new group of broad-spectrum antimicrobials. Here, using a set of in vivo experiments, we assessed the potential of ceragenins in the eradication of an important etiological agent of nosocomial infections, *Acinetobacter baumannii*. Assessment of the bactericidal effect of ceragenins CSA-13, CSA-44, and CSA-131 on clinical isolates of *A. baumannii* (*n* = 65) and their effectiveness against bacterial cells embedded in the biofilm matrix after biofilm growth on abiotic surfaces showed a strong bactericidal effect of the tested molecules regardless of bacterial growth pattern. AFM assessment of bacterial cell topography, bacterial cell stiffness, and adhesion showed significant membrane breakdown and rheological changes, indicating the ability of ceragenins to target surface structures of *A. baumannii* cells. In the cell culture of A549 lung epithelial cells, ceragenin CSA-13 had the ability to inhibit bacterial adhesion to host cells, suggesting that it interferes with the mechanism of bacterial cell invasion. These findings highlight the potential of ceragenins as therapeutic agents in the development of antimicrobial strategies against bacterial infections caused by *A. baumannii*.

## 1. Introduction

Due to the increasing number of infections caused by bacteria with various resistance mechanisms, there is an urgent need to conduct research aimed at the synthesis of new antibiotics. An important area of research in this field is the search for natural antimicrobial peptides and the synthesis of their improved mimics. Ceragenins are an example of such a group of compounds. Although ceragenins are non-peptide, cholic acid-based compounds, they imitate the action of antimicrobial peptides and exhibit a wide spectrum of antimicrobial effects [1,2,3]. The mechanism of action of ceragenins is determined by the positive charge, which, through physical interactions with the negatively charged cell membrane of microorganisms, results in ceragenin incorporation into the bilayer structure, followed by its disruption and microorganism death. The mechanism of action can be explained by the “carpet” hypothesis, which suggests that antimicrobial molecules rupture the bacterial membrane and produce micelles due to the tension generated by the antimicrobial once it reaches its critical concentration on the membrane surface [4]. Moreover, ceragenins were reported to bind to lipopolysaccharide (LPS) and lipoteichoic acid (LTA) of Gram-negative and Gram-positive bacteria, and for this reason they inhibit innate immune recognition. A mechanism of membrane disruption based on the production of reactive oxygen species has also been described [5]. These compounds show low potential for toxic effects against host cells at concentrations of effective bactericidal activity, making them relatively safe substances [6,7]. Due to the much simpler structure, the cost associated with the ceragenins’ synthesis is much lower compared to AMPs or their amino acid-based mimics. This is an important feature in terms of the possibility of using ceragenins on a large scale. Additionally, ceragenins as cholic acid derivatives are not enzymatically inactivated by proteases, retaining their activity at bacterial infection sites in proteolytic enzymes released mostly from neutrophils [6]. Ceragenins show synergistic or additive effects with conventional antibiotics or with other ceragenins. It is noteworthy that, so far, an antagonistic effect was not observed [7,8]. An important feature of ceragenin is its anti-biofilm activity—both inhibiting its formation (preventive) and eradicating bacterial cells in pre-form biofilm mass (curative) [9]. The possibility of resistance generation to ceragenins is small compared to conventionally used antibiotics. Bacteria are able to develop adaptive resistance mainly through modification of lipid A, while in the absence of ceragenins’ effects on their cells, they revert to susceptible forms [10].

On a global scale, the clinical significance of this pathogen results from its outstanding ability to develop and acquire various mechanisms of antibiotic resistance [11]. These aerobic, pleomorphic, and non-motile bacteria are resistant to all β-lactams, including carbapenems, and display a strong ability to rapidly change under selective environmental pressures [12]. In most cases, a significant proportion of *A. baumannii* clinical isolates originated from respiratory samples of hospitalized patients, and this etiology is of particular importance in the individual diagnosis of ventilator-associated pneumonia (VAP) in the ICU [13,14,15]. *A. baumannii* is equipped with various virulence factors, including LPS, siderophore-mediated iron acquisition systems, and factors regulating biofilm formation and adhesion [16]. The ability of *A. baumannii* to adhere and form biofilms on various surfaces (pili formation and exopolysaccharide production, respectively) largely determines its importance in the hospital setting [17,18]. Interestingly, its adherence to host cells can cause Omp38-mediated apoptosis [19]. Here, we present experimental evidence from in vitro studies demonstrating the potent bactericidal activity of ceragenins against various clinical isolates of *A. baumannii*, the ability of ceragenins to combat *A. baumannii* biofilm on abiotic surfaces, and the ability to prevent its attachment to host cell surfaces.

## 2. Results

### 2.1. Ceragenins CSA-13 and CSA-131 Showcase Potent Antibacterial Efficacy against A. baumannii and CRAB Strains

**Figure 1:**
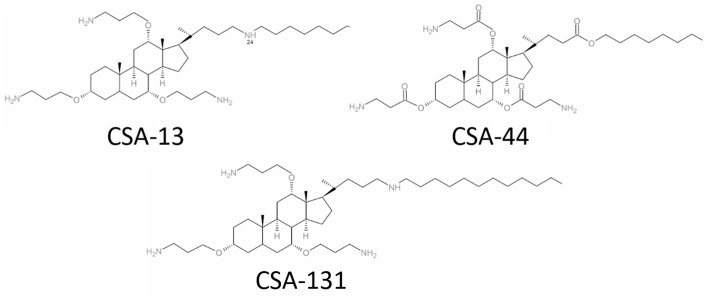
Structural formulae of ceragenins CSA-13, CSA-44, and CSA-131.

**Figure 2:**
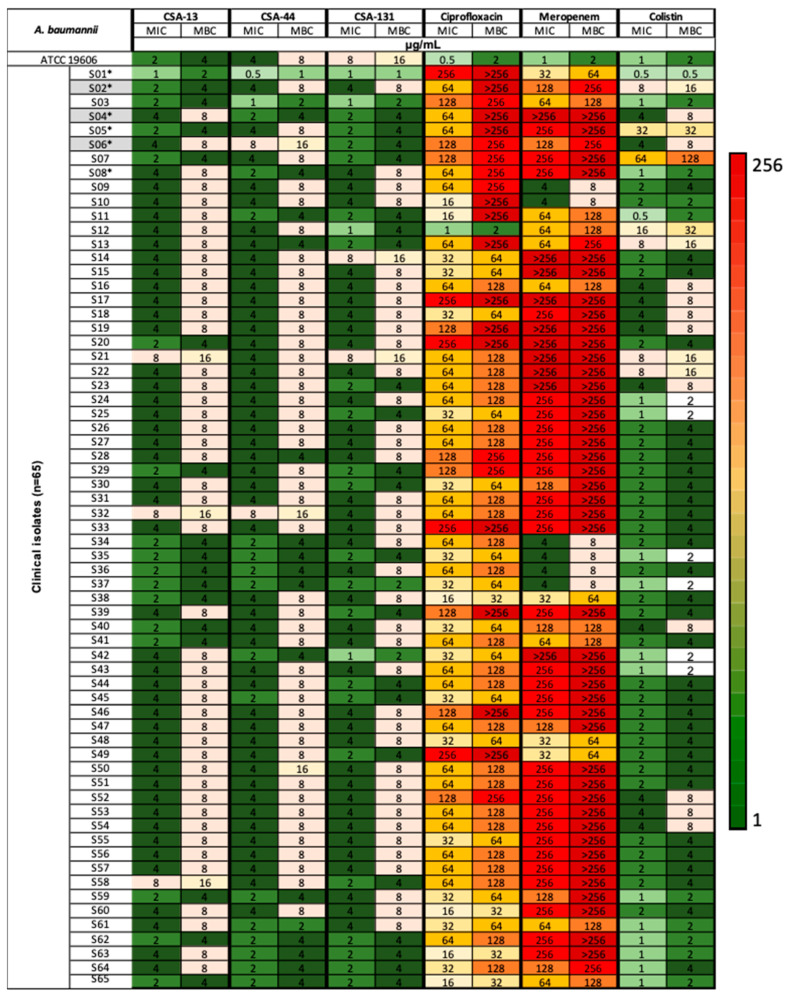
Minimal inhibitory concentrations (MICs) and minimal bactericidal concentrations (MBCs) of ceragenins (CSA-13, CSA-44, and CSA-131) and conventional antibiotics (ciprofloxacin, meropenem, colistin) against laboratory (ATCC 199606) and clinical strains of *A. baumannii* (*n* = 65). * Indicates the carbapenem-resistant *A. baumannii* strains tested here in other experimental settings.

The microdilution method was used to evaluate the MIC and MBC of ceragenins (CSA-13, CSA-44, and CSA-131; Figure 1) and conventional antibiotics (ciprofloxacin, meropenem, and colistin) against a single laboratory strain and 65 clinical strains of *A. baumannii* (Figure 2).

As demonstrated, the MIC values of laboratory strain ATCC 199606 range between 0.5 and 1 µg/mL, and the MBC is 2 µg/mL, showing a high level of susceptibility to ciprofloxacin, meropenem, and colistin. When analyzing the antibiotic susceptibility of clinical isolates, it was noted that the MIC values for ciprofloxacin ranged in a majority of cases from 16 to 256 µg/mL, while one clinical strain had a significantly low MIC value of 1 µg/mL, which indicates a high susceptibility to ciprofloxacin. In the same way, the MBC for these bacteria was found to be 2 µg/mL, showing that it can kill bacteria effectively at a low dose. There was a range of MIC values for meropenem of 4–256 µg/mL. Most bacteria had MBC values higher than 256 µg/mL. Colistin demonstrated a range of MIC values of 0.5–64 µg/mL, with MBC values for a majority of strains ranging from 1 to 16 µg/mL.

In contrast to the variable susceptibility of isolates to conventional antibiotics, ceragenins had effective antibacterial action against the majority of tested strains. The MIC values of tested ceragenins (CSA-13, CSA-44, and CSA-131) for carbapenem-resistant *A. baumannii* strains (S01, S02, S04, S05, S06, and S08) were assessed. MIC values of 1–4 µg/mL for CSA-13, 0.5–8 µg/mL for CSA-44, and 1–4 µg/mL for CSA-131 were observed. Remarkably, these values were found to be in alignment with the low MICs observed for *A. baumannii* strains not resistant to carbapenems. In detail, the MIC values for ceragenin CSA-13 ranged from 1 to 4 µg/mL, with most strains showing MIC values within this range. Importantly, among the 66 isolates tested, only three strains exhibited elevated MIC values of 8 µg/mL, suggesting a lower susceptibility to CSA-13. Similarly, the strains exhibited MBC values of 16 µg/mL. Similarly, the CSA-44 displayed a range of MIC values, varying from 0.5 to 4 µg/mL for a majority of strains; only two strains had higher MIC/MBC values of 8/16 µg/mL. Typically, CSA-131 showed MBC values between 1 µg/mL and 16 µg/mL and MIC values between 1 µg/mL and 8 µg/mL. Overall, the low MIC values of the ceragenins demonstrated a considerable efficacy in inhibiting bacterial growth against a broad spectrum of *A. baumannii* strains, including those present in clinical environments.

**Figure 3:**
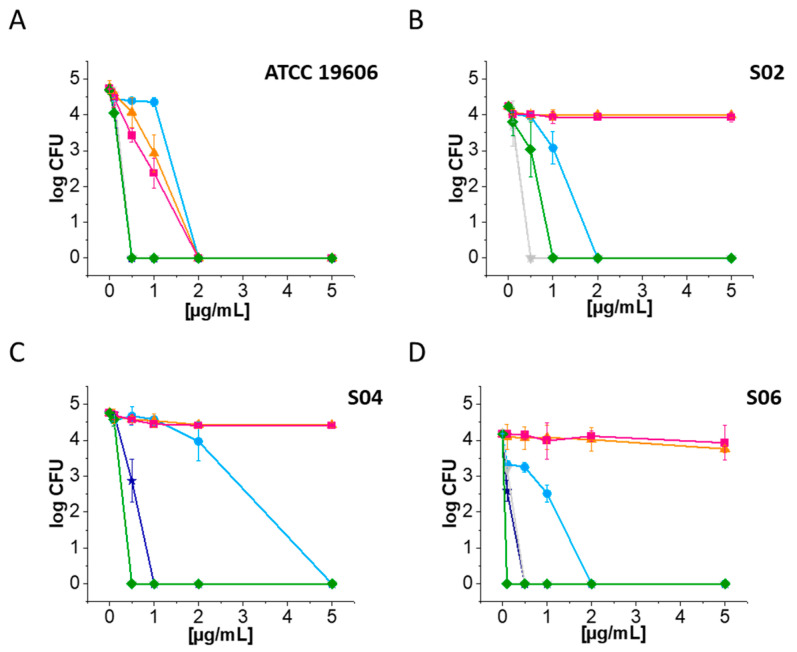
Bactericidal activities of ceragenins (CSA-13, dark blue stars; CSA-44, blue circles; CSA-131, grey triangles) and conventional antibiotics (ciprofloxacin, orange triangles; meropenem, pink squares; colistin, green rhombus) against *A. baumannii* (ATCC 19606) (**A**) and clinical strains of carbapenem-resistant *A. baumannii* (S02, (**B**); S04, (**C**); S06, (**D**)) determined with use of colony counting assay (killing assay).

In order to assess the bactericidal effects of ceragenins and conventional antibiotics on CRAB strains, we employed a standard bacteria-killing assay experiment. The susceptibility data obtained from our research clearly indicate that ceragenins exhibit more potent bactericidal activity against CRAB strains compared to standard antibiotics such as ciprofloxacin and meropenem (Figure 3).

Furthermore, CSA-13 and CSA-131 exhibited significantly greater efficacy against *A. baumannii* strains (Figure 3A–D), within the concentration range of 0.5–1 µg/mL. Conversely, no significant bactericidal effect was noted for CRAB strains after treatment with ciprofloxacin and meropenem, even at a concentration of 5 µg/mL (Figure 3A–D).

### 2.2. Atomic Force Microscopy Measurements of A. baumannii Cells Subjected to Deragenin Addition

**Figure 4:**
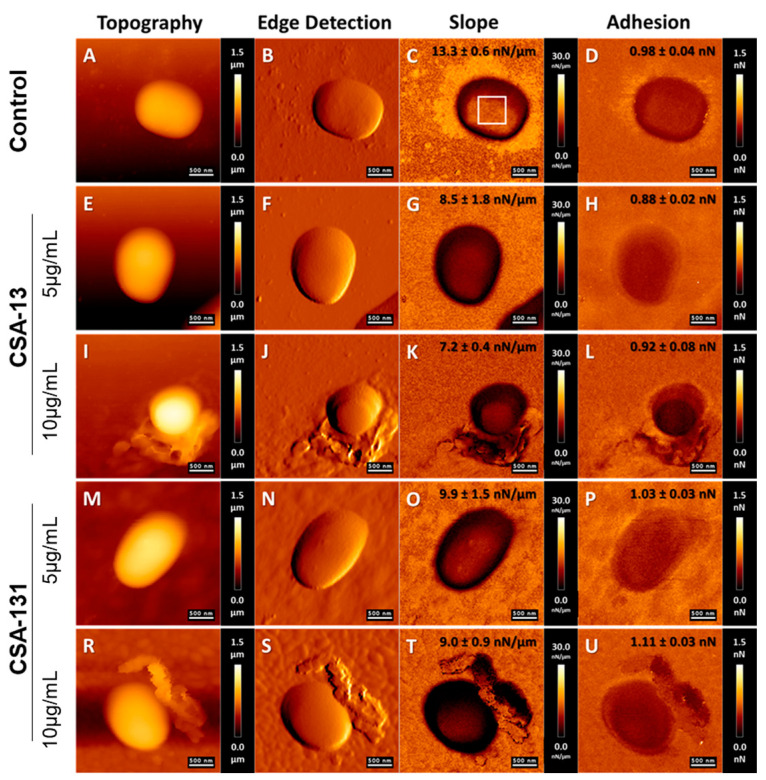
Atomic force microscopy measurements (JPK QI™ mode—Quantitative Imaging) of carbapenem-resistant *A. baumannii* cells (clinical strain No. 2) subjected to ceragenin treatment. Examination of AFM topography, cell stiffness (Slope), and adhesion upon addition of CSA-13 and CSA-131 at 5 µg/mL and 10 µg/mL, respectively.

Visualization of the morphological alteration of *A. baumannii* cells subjected to ceragenins CSA-13 and CSA-131 using AFM is shown in Figure 4.

An AFM study was performed to confirm the antibacterial effect of selected ceragenins on *A. baumannii* cells and the destruction of cell membranes caused by ceragenins. Figure 4 presents the representative morphology of bacterial cells after CSA-13 and CSA-131 treatment. The obtained results indicate that the mechanism of ceragenins action consists of their interaction with the bacterial membrane. Morphological alterations in bacterial *A. baumannii* cells subjected to ceragenin CSA-13, and CSA-131, i.e., microcracks, were observed compared to the untreated cells, especially at a dose of 10 µg/mL (Figure 4I,R). AFM analysis showed no significant change in the morphology of the treated bacteria using ceragenins at a lower dose of 5 µg/mL. In the case of CSA-13 treatment, the shape of the bacteria became more oval (Figure 4I). The stiffness of the bacterial surface decreased upon tested ceragenin addition. In the case of CSA-13 (at a dose of 10 µg/mL), the stiffness of bacterial cells decreased by 45.9% compared to the untreated cells; in the case of CSA-131 treatment (at a dose of 10 µg/mL), the stiffness decreased by 32.3%. The results indicate that changes in the of membrane lipid organization may be related to changes in the mechanical properties of cell surfaces. In case of CSA-13 treatment (at a maximum dose of 10 µg/mL), the adhesion forces of bacterial surfaces decreased by 6.1%. In the case of CSA-131, the adhesion forces increased by 13.3% (Figure 4U).

### 2.3. Biofilm Eradication of Carbapenem-Resistant A. baumannii after CSA-13 Addition at Low Concentration

**Figure 5:**
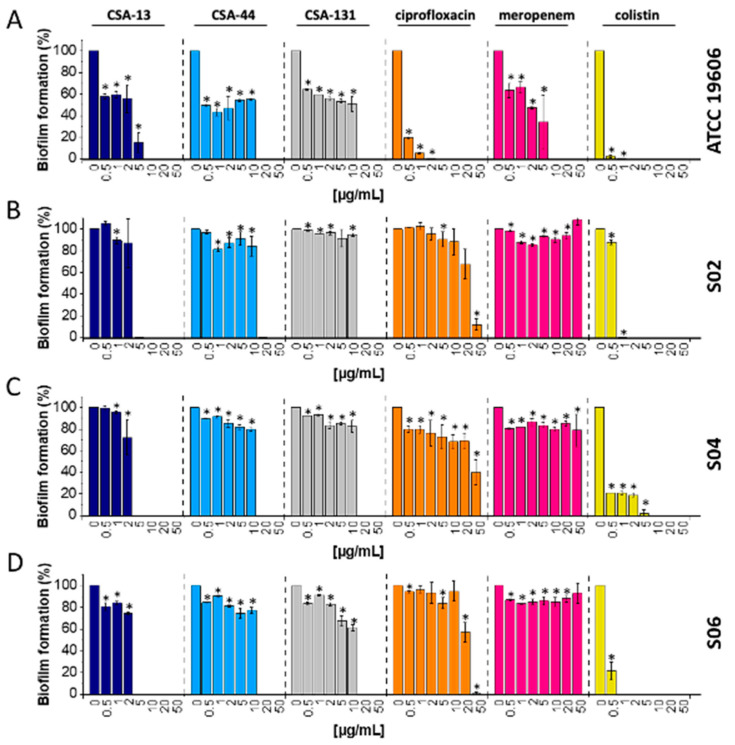
Anti-biofilm effects of ceragenins and conventional antibiotics against *A. baumannii*. Increasing concentrations (0–50 µg/mL) of ceragenins (CSA-13, dark blue columns; CSA-44, blue columns; CSA-131, grey columns) and conventional antibiotics (ciprofloxacin, orange columns; meropenem, pink columns; colistin, yellow columns) were used to treat laboratory (ATCC 19606, (**A**)) and clinical strains of carbapenem-resistant *A. baumannii* (S02, (**B**); S04, (**C**); S06, (**D**)). After treatment, the remaining biofilm was stained with resazurin to assess its viability. The results are presented as mean ± SD. * Indicates statistical significance (*p* < 0.05) when compared to control.

The results of our study indicate that CSA-13 showed the highest level of antibiofilm activity against both the laboratory strain and carbapenem-resistant *A. baumannii* (S02, S04, S06) that were evaluated. Significantly, the complete elimination of biofilm was observed at a concentration of 5 μg/mL of CSA-13 or 20 µg/mL of CSA-44 or CSA-131. It is important to note that all ceragenins were better at preventing biofilm formation than ciprofloxacin and meropenem (Figure 5A–D), and only colistin had comparable antibiofilm properties to CSA-13. 

The results show that CSA-13 has promising antibiofilm qualities and could be a very effective way to treat CR *A. baumannii* infections. Furthermore, the great effectiveness of CSA-44 and CSA-131 in eliminating *A. baumannii* biofilms suggests that they may be useful substitutes for other therapies. 

### 2.4. Ceragenins Exhibited Significant Potential as Antibiofilm Agents against A. baumannii Forming Biofilm on Tracheostomy Tube Surface

**Figure 6:**
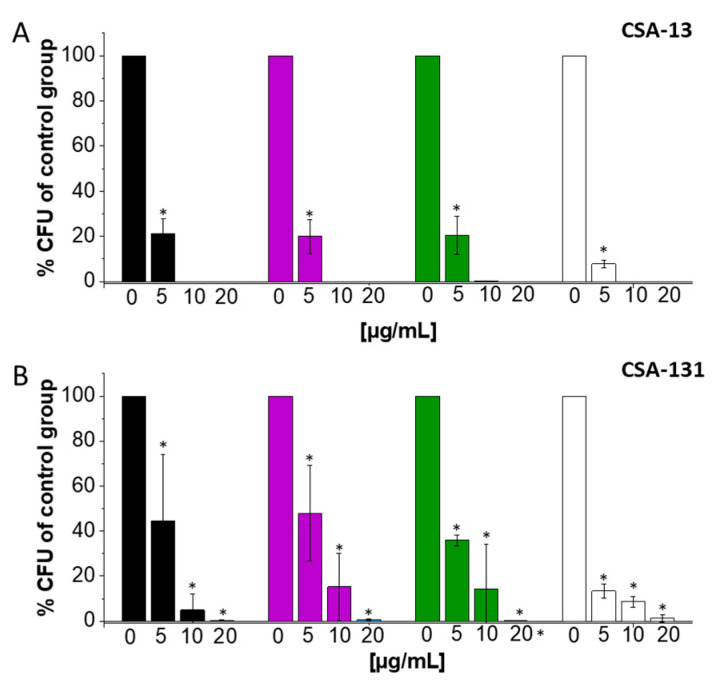
Decrease of *A. baumannii* cell viability in the biofilm pre-formed on the surface of silicone biomedical tubes after CSA-13 (**A**) and CSA-131 (**B**) treatment. The percentage of biofilm-embedded bacteria for laboratory strain (ATCC 19606, black columns) and carbapenem-resistant clinical strains of *A. baumannii* (S02, purple columns; S04, green columns; S06, white columns) was estimated after sonication of the preformed biofilm attached to the surface in the final moment of exposure to the tested ceragenin. The results are presented as mean ± SD. * Indicates statistical significance (*p* < 0.05) when compared to control.

As anticipated, *A. baumannii* grows in biofilm formed on silicone endotracheal tube surfaces. The application of ceragenins (CSA-13 and CSA-131) to such a culture revealed strong antibiofilm activity against CR *A. baumannii* cells. More precisely, at a concentration of 10 µg/mL, CSA-13 effectively eradicated bacterial cells within biofilm on the tested tube segments (Figure 6A). In addition, the application of 5 µg/mL of CSA-13 led to a decrease in biofilm by about 80% or more in the case of laboratory and clinical strains of *A. baumannii*. Additionally, the application of CSA-131 showed a concentration-dependent antibiofilm effect on silicone tube biofilm growth (Figure 6B). Its effectiveness was found to be marginally less than that of CSA-13, though. However, CSA-131 demonstrated a noteworthy decrease in biofilm eradication even at reduced concentrations, indicating its promise as an antibiofilm agent against *A. baumannii* on tracheostomy tubes.

### 2.5. Ceragenins Display Low Cytotoxicity against A549 Cells at Bactericidal Concentrations

**Figure 7:**
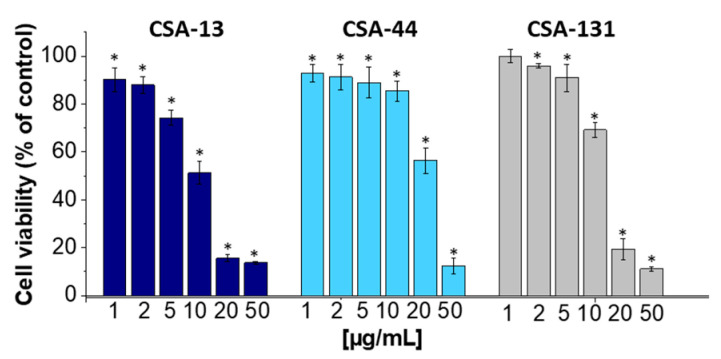
Viability of lung epithelial A549 cells upon 24 h exposure to ceragenins (CSA-13, dark blue columns; CSA-44, blue columns; CSA-131, grey columns) at doses ranging from 1 to 50 µg/mL. The viability of cells was determined using MTT assay. The results are presented as mean ± SD. * Indicates statistical significance (*p* < 0.05) when compared to control.

The MTT assay was used to evaluate the cytotoxicity of ceragenins towards A549 epithelial cells. As demonstrated, CSA-13, CSA-44, and CSA-131 displayed dose-dependent effects. To be more precise, ceragenins significantly reduced cell viability at higher concentrations (20 µg/mL and 50 µg/mL) as compared to the control group (Figure 7). On the other hand, CSA-44 and CSA-131 exhibited a mild decline in cell viability at higher concentrations (beyond 20 µg/mL), with less noticeable cytotoxicity.

### 2.6. Anti-Adhesive Properties of CSA-13

**Figure 8:**
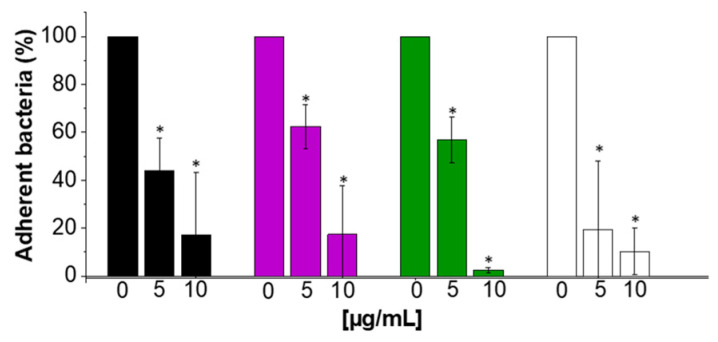
The impact of ceragenin CSA-13 on the adherence of the laboratory strain of *A. baumannii* (ATCC 19606, black columns) and clinical strains of carbapenem-resistant *A. baumannii* (S02, purple columns; S04, green columns; S06, white columns) to lung epithelial A549 cells. The adherence of bacterial cells to A549 was determined after 2 h exposure. The results are presented as mean ± SD. * Indicates statistical significance (*p* < 0.05) when compared to control.

The adhesion assay performed using the A549 cell line revealed that CSA-13 significantly affected the laboratory strain ATCC19606 and carbapenem-resistant *A. baumannii’s* (S02, S04, S06) adherence to the host cells (Figure 8). The effectiveness of CSA-13 in inhibiting bacterial adhesion increased proportionally to its concentration. At different doses, CSA-13 exhibited a significant inhibitory effect, with the most prominent effect at 10 μg/mL, where bacterial adhesion was, on average, 11% lower upon CSA-13 treatment compared to the untreated control. These results highlight how well CSA-13 inhibits *A. baumannii* attachment to epithelial cells, indicating that CSA-13 may be used as a therapeutic drug to stop carbapenem-resistant *A. baumannii* cell invasion.

## 3. Discussion

Carbapenem-resistant *A. baumannii* (CRAB) causes severe, often fatal nosocomial infections, and for this reason it is classified as a priority pathogen 1 (critical) on the World Health Organization’s (WHO) list of priority pathogens for research and development of new antibiotics [20]. Very few antimicrobials available for clinical use are effective against it—in monotherapy, sulbactam is one drug used to treat *A. baumannii*; most other treatment options include the combination of different medications with sulbactam [21,22,23,24]. For this reason, there is a need for the advancement or revelation of novel treatments, meticulously conducted clinical studies of current antimicrobial treatment strategies, and combinations to limit the spread of MDR *A. baumannii* infection in healthcare settings. Phage therapy and small molecules are two potential treatments for CRAB that are currently under investigation [25,26]. Moreover, the antibacterial alternatives available to CRAB are severely limited by acquired and inherent resistance mechanisms [24,27,28]. For multidrug-resistant *A. baumannii* (MDR-AB), the colistin (Polymyxin E; produced by *P. polymyxa* var. *colistinus*) is presently used as an initial treatment option [29]; however, due to the negative consequences of nephrotoxicity and the increasing incidence of resistance, its use poses significant challenges [30]. The primary objective of our study was to assess the efficacy of ceragenin in combating *A. baumannii* infection, particularly carbapenem-resistant *A. baumannii*. The results unambiguously demonstrate the favorable potential of ceragenins for clinical utilization against *A. baumannii* infections.

An investigation into the antibacterial properties against 65 strains of *A. baumannii* revealed that ceragenins have remarkable antimicrobial efficacy, frequently surpassing that of traditional antibiotics like ciprofloxacin and meropenem, particularly when combating CRAB strains. The unique thing about our study is that we tested the antibacterial abilities of ceragenins against a large group of clinical strains of *A. baumannii*. Our findings are consistent with previous research demonstrating the effectiveness of CSA-13 and CSA-131 against isolates of *A. baumannii* that are resistant to both colistin and carbapenem [31]. Additionally, it was shown that, with a MIC_50_ value of 2 µg/mL, CSA-13 and CSA-131 were effective against resistant *A. baumannii* [31]. The findings from the killing assay study demonstrate that both CSA-13 and CSA-131 show substantial antibacterial efficacy against the laboratory strain and all the clinical strains of *A. baumannii* that were evaluated. Moreover, one noteworthy advantage of ceragenins is that they are soluble in water, in contrast to many antimicrobial peptides (AMPs), which precipitate in aqueous solutions. This characteristic guarantees that ceragenins will keep their antibacterial effectiveness without aggregation-related problems. Thus, the solubility of ceragenins represents a significant advancement in the development of AMP-mimetic compounds, providing a more reliable and effective therapeutic option. 

Our study’s uniqueness consists of utilizing atomic force microscopy to record bacterial images in live settings and measure physical alterations in bacterial cells of *A. baumannii* after ceragenin addition. This methodology improves traditional microbiological testing by providing a more thorough understanding of how tested antibiotics carry out their bactericidal effects. Moreover, our data about the mechanical characteristics of treated cells provide an understanding of the biophysical impacts of ceragenins, which could aid in developing and enhancing these drugs for therapeutic use. Several AFM investigations have examined the impact of antibiotics, such as antimicrobial peptides, on the bacterial surface [32,33,34]. This research, conducted over time, has significantly enhanced our understanding of the mechanisms by which different antibiotics work. The study demonstrated that exposure to CSA-13 and CSA-131 at doses of 5 and 10 µg/mL caused damage to the outer membrane of *A. baumannii* cells, resulting in observable alterations in the topography of CR *A. baumannii* cells. This is confirmed by the results in [35], where the effect of selected ceragenins on the morphology of *Klebsiella pneumoniae* BAA-2473 cells was studied. Interaction between ceragenins and bacterial membrane affects membrane molecule rearrangement, leading to its disruption. Consistent with prior hypotheses that suggest AFM should be regarded as a precise instrument for assessing pathogens’ susceptibility to antimicrobial agents, our investigations demonstrated the pathogens’ membrane damage, which was further accompanied by intracellular content leakage or even complete cell lysis [36]. Our study showed that both CSA-13 and CSA-131 resulted in substantial decreases in cell stiffness. These results strongly suggest that ceragenins induce substantial alterations in the arrangement of membrane lipids, leading to modifications in the mechanical characteristics of the bacterial cell surface. CSA-13 exhibits a more significant decrease in stiffness, suggesting that it has the potential to produce a more noticeable disruption in the integrity of the membrane when compared to CSA-131. In addition, our data uncovered a distinct impact of ceragenin on the bacterial ability to adhere to surfaces. Administration of CSA-13 resulted in a 6.1% drop in adhesion forces, suggesting a decline in the capacity of bacterial cells to attach to surfaces. This could be advantageous in the prevention of biofilm development. Conversely, the addition of CSA-131 resulted in a 13.3% increase in adhesion forces, indicating a distinct stage of interaction with the bacterial membrane. The observed increase in adhesion forces may be attributed to the reorganization of membrane components, which enhance surface binding capabilities, in agreement with our previous observation that bacteria, upon ceragenins addition, undergo different stages, from increasing bacteria wall stiffness at the initial stage to its decrease later, when morphological manifestation of bacterial cell damage is visible [37]. These findings add to the increasing amount of evidence that supports the therapeutic potential of ceragenins against antibiotic-resistant bacteria, addressing an important requirement in contemporary healthcare.

A cellular adaptation known as bacterial biofilm development enables pathogens to endure in hostile environments, especially by fortifying their resistance to conventional antibiotic treatment [38]. The unique characteristics of ceragenins demonstrate their promise as a successful strategy for treating bacterial infections like CR *A. baumannii*. The demonstrated abilities of *A. baumannii* strains to form biofilms on both living and non-living surfaces, which shields them from antibacterial and antiseptic treatments as well as the host immune response, are particularly noteworthy. Undoubtedly, the capacity of *A. baumannii* to thrive as a biofilm can potentially enhance its endurance in the hospital setting, hence elevating the probability of nosocomial infections and outbreaks. Our research also highlights CSA-13’s remarkable antibiofilm action. At a low dosage of 5 µg/mL, CSA-13 completely eradicated carbapenem-resistant *A. baumannii* biofilms; greater concentrations of CSA-44 and CSA-131 (20 µg/mL) were required to obtain comparable outcomes (Figure 5). Comparing CSA-13 to other ceragenins, the data collected indicate that CSA-13 is particularly effective at preventing and disrupting biofilm formation at lower concentrations. Other works also present similar data with different strains of bacteria and fungi [39,40]. It is remarkable that CSA-13 was just as effective as colistin, which is well-known for having potent antibiofilm capabilities, at preventing the formation of biofilms.

The results proving the antibiofilm characteristics of ceragenins in medical applications fill a vital gap in the management of infections related to medical equipment. Especially remarkable is the ability of ceragenins to disrupt biofilm development on tracheostomy tubes [41]. Clinically, biofilm on medical devices is a major concern because of its importance in chronic infections and antibiotic resistance. *A. baumannii‘s* potential for pathogenicity is dictated by multiple characteristics, including as putative virulence factors like outer membrane protein A (OmpA), lipopolysaccharides, K1 capsular polysaccharide, and external appendages like pili [42,43,44]. Moreover, *A. baumannii’s* capacity for virulence has been linked to plasmids carrying genes for organic peroxide resistance [45,46]. One of the most persistent and antibiotic-resistant infections is due in large part to its improved capacity to form biofilms and its ability to colonize and survive on a wide variety of biotic and abiotic surfaces [47]. Quorum sensing (QS) is critical for the production of biofilms in *A. baumannii*, which is a mechanism necessary for the survival, pathogenicity, and resistance to antibiotics of the bacteria [48,49,50,51]. The immune system and antibiotics are just two of the hostile environments that bacteria are protected from by biofilms. *A. baumannii*’*s* QS system uses signaling molecules such acyl-homoserine lactones (AHLs) to coordinate the expression of genes involved in the creation and maintenance of biofilms [52]. One proposed mechanism by which ceragenins may exert their antibacterial and anti-adhesive properties is by disrupting quorum sensing signals. Ceragenins may potentially hinder the communication process mediated by AHL, impeding the coordinated behavior necessary for creating and maintaining biofilms. This interference might be caused by the direct binding of the substance to QS molecules or their receptors, inhibiting the pathways responsible for signal transduction. Consequently, ceragenins cause a decrease in the production of biofilms and an increase in the vulnerability of bacteria to environmental pressures and antimicrobial substances.

The Csu pili in *A. baumannii* play a crucial role in the production of biofilms on both abiotic surfaces, like medical equipment, and biotic surfaces, like epithelial cells [53]. The Csu pili also enhance virulence by facilitating bacterial adhesion, a critical process for colonization and infection [54]. The regulation of Csu pili expression is governed by a complex network that involves c-di-GMP signaling and certain regulatory proteins such as PdeB [55]. These proteins play a role in controlling the formation and activity of pili. Ceragenins might have the potential to interfere with the formation or operation of Csu pili, hence impeding the capacity of *A. baumannii* to attach to surfaces and create biofilms. This interruption could arise from various mechanisms: (i) direct interaction via binding of ceragenins directly to the pili or their subunits, preventing proper assembly or function; and (ii) interfering with the regulatory pathways controlling pili expression, such as the c-di-GMP signaling network or PdeB activity. Ceragenins may have the potential to impact these pathways, resulting in a decrease in the generation or effectiveness of pili. By affecting these pathways, ceragenins could reduce the production or functionality of pili, leading to diminished adhesion and biofilm formation.

Our findings indicate that CR *A. baumannii* was able to easily build biofilms on silicone tube segments, indicating the biomedical surfaces’ vulnerability to bacterial colonization (Figure 6). On the other hand, ceragenin presence successfully eradicates biofilm, indicating the potential as a medical device protection agent. Our observation is in great agreement with previous reports. For example, Pollard et al. described the efficacy of CSA-13 in eliminating both Gram-positive and Gram-negative biofilms at concentrations comparable to ciprofloxacin while still killing drug-resistant pathogens [56]. Moreover, ceragenins have been effectively used in fracture fixation plates and contact lenses to stop and eradicate *Pseudomonas aeruginosa* and *Staphylococcus aureus* biofilms, demonstrating their adaptability in a range of medical settings [57]. The versatile characteristics of ceragenins, including strong antibacterial and antibiofilm properties, high stability in body fluids, and physical properties, make them valuable candidates for medical applications. Thin films containing CSA-131 may protect endotracheal tubes from microbial colonization, preventing infection and irritation linked to intubation, according to recent research by Hashemi et al. [58]. 

The potential, safe therapeutic application of ceragenins is indicated by the fact that the doses needed for bactericidal action against *A. baumannii* are within well-recognized biocompatibility ranges. Since the A549 cell line used in our study is a human lung cancer cell line, cytotoxicity with ceragenins may be higher than with primary cell lines because both AMPs and ceragenins are thought to be anticancer agents because they exhibit increased cytotoxic activity against transformed cells like cancer cells [59,60]. Cancer cells have a different membrane makeup than primary, non-transformed cells. As such, data on the cytotoxicity of ceragenins may not be entirely reliable when derived from experiments conducted on transformed cancer cells. Research utilizing primary cells might yield more accurate cytotoxicity measurements. However, these investigations using the A549 cell line reveal details regarding a minimum window of activity against bacteria that does not cause damage to eukaryotic cells. Particular consideration should be taken regarding CSA-13’s anti-adhesive qualities. Ceragenin CSA-13 considerably reduced the adhesion of laboratory strain ATCC19606 and carbapenem-resistant *A. baumannii* (S02, S04, S06) to host cells, according to an adhesion assay using the A549 cell line. Bacterial adherence was inhibited concentration-dependently; the greatest effect was seen at 10 µg/mL, at which point bacterial adhesion was around 11% less than in the control. These results show how CSA-13 may be explored in developing new methods to treat carbapenem-resistant *A. baumannii* infections by preventing the microorganism from attaching to epithelial cells, an essential stage in the infection process.

## 4. Materials and Methods

### 4.1. Clinical Bacterial Isolates and Antimicrobial Compounds

The study utilized a laboratory strain of *A. baumannii* was purchased from American Type Culture Collection (ATCC 19606, USA) and 65 clinical strains of *A. baumannii* isolated from the bronchial lavage, sputum, throat swab, and swab from a tracheostomy wound that tested positive in conventional diagnostic microbiology laboratories. The isolates were cultured on agar plates and analyzed using the VITEK^®^ 2 system (bioMérieux, Marcy-l’Etoile, France). Ceragenins, namely CSA-13, CSA-44, and CSA-131, were synthesized using the method reported previously [14]. Colistin (#C4461), ciprofloxacin (#17850), and meropenem (#PHR1772) from Sigma Aldrich (USA) were the conventional, control antibiotics used in this study. All agents were dissolved in sterile saline and stored at a temperature of 4 °C until the experiments were conducted. Sterile endotracheal tubes were sourced from Zarys company (Poland).

### 4.2. Mechanisms of Resistance of the Tested Clinical Strains of A. baumannii

Strains S01 (S-strain; 02–number 2), S02, S04, S05, S06 and S08, isolated from sputum, were identified as carbapenem-resistant *A. baumannii* (CRAB) strains were tested using a combination disc assay, following the guidelines outlined by the European Committee on Antimicrobial Susceptibility Testing (EUCAST; www.eucast.org) (accessed on 12 May 2023).

### 4.3. Antimicrobial Activity Assessment

The minimum inhibitory concentration (MIC) and minimum bactericidal concentration (MBC) of ceragenins (CSA-13, CSA-44, and CSA-131) and conventional antibiotics (colistin, ciprofloxacin, and meropenem) were assessed against laboratory (*n* = 1) and clinical strains (*n* = 65) of *A. baumannii* and carbapenem-resistant *A. baumannii* in Muller–Hinton broth using the microdilution method with concentrations of the drugs varied between 0.125 µg/mL and 256 µg/mL. The lowest concentration of ceragenins and antibiotics that, during an overnight culture, produced no discernible growth was known as the MIC. Each dilution series of the overnight MIC had samples quantitatively plated on agar to obtain the MBC values. At least 99.9% of the viable counts were reduced by the MBC, the lowest concentration of antimicrobial agents. In another series of tests, the killing assay method was used to investigate the capacity of treated carbapenem-resistant *A. baumannii* (S02, S04, S06) cells to form colonies after incubation with investigated substances at doses between 1 and 5 µg/mL. Following a 1 h incubation at 37 °C, the suspensions were diluted 10- to 1000-fold in PBS, the plates were placed on ice, and 10 μL aliquots were plated on MH agar to measure the CFUs. Cell survival was expressed upon overnight culture when compared to untreated controls (0 µg/mL).

### 4.4. Atomic Force Microscopy Measurements of A. baumannii Cells Subjected to Ceragenin Treatment

*A. baumannii* carbapenem-resistant strains (S02) grown on MacConkey agar were resuspended in PBS (OD600: ~ 0.1), followed by incubation with 5 µg/mL and 10 µg/mL of CSA-13 and CSA-131 ceragenins. Next, 200 µL of bacterial samples was transferred onto the mica surface previously functionalized with Poly-L-lysine; −100 μL of 0.01% Poly-l-lysine solution (Sigma-Aldrich, #25988-63-0) was incubated with cut mica slices. The attachment of bacterial cells to the mica surface was achieved during a 0.5 h incubation. Images of bacterial cells and characterization of their mechanical properties were collected using a NanoWizard 4 BioScience atomic force microscope (AFM–JPK/Bruker, MA, USA) equipped with a liquid cell setup. Because of the lateral forces occurring during data collection, which may have caused cells to move across the surface, the contact mode scanning AFM was operated in QI mode (Bruker/JPK QI™ mode—Quantitative Imaging). Silicon nitride AFM cantilevers (Bruker, MSCT-A) had a nominal spring constant of 0.07 N/m and measured spring constant in the range of 0.067–0.099 N/m, using the thermal tune method. The bacterial cells were located using 20 × 20 μm maps; then, topography maps with a size of 3 μm × 3 μm were created, with a resolution of 128 pixels per line, in wet conditions. QI maps were used to determine bacteria surface stiffness (JPK Slope Mode) and adhesion forces between the cells and the AFM probe (JPK Adhesion Mode). The rigidity of bacteria cells was calculated based on force–indentation curves from QI maps. The slope of the force curves was recorded on mica, and bacterial surfaces were fitted using linear regression and used as a stiffness parameter (nN/μm) reflecting bacterial stiffness.

### 4.5. Activity against Biofilms

The effectiveness of ceragenins (CSA-13, CSA-44, and CSA-131) and conventional antibiotics (colistin, ciprofloxacin, and meropenem) to prevent biofilm formation was investigated by incubating carbapenem-resistant *A. baumannii* (S02, S04, S06) with varying doses (1–50 μg/mL) of the studied compounds for 72 h at 37 °C. An overnight pathogen culture was diluted to 10^5^ CFU/mL before the antimicrobial exposition. After the incubation period, the planktonic bacteria-containing growth media was removed, and the wells were gently washed three times with PBS. To stain the biofilm, resazurin at a final concentration of 200 µg/mL was utilized and left for further incubation for 1h. To ascertain the viability of biofilms, fluorometric analysis (λ = 520/590 nm) was employed.

### 4.6. Evaluation of Ceragenins’ Antibiofilm Capabilities on Tracheostomy Tubes

Sterile culture tubes were utilized to place 50 mm segments of silicone tracheostomy tubes. Inoculation solutions containing 10^6^ CFU/mL of laboratory and carbapenem-resistant *A. baumannii* (S02, S04, S06) were prepared and used to inoculate the tracheostomy segments in a volume of 1.5 mL. CSA-13 and CSA-131 were then introduced at doses ranging from 0 to 20 µg/mL. After 24 h, the tracheostomy segments were removed, gently washed with sterile PBS, and sonicated for 15 min using a bath sonicator to detach the bacteria adhered to the tubes’ surface. Colonies were counted after the resultant solutions were serially diluted, plated on MH agar, and incubated for 24 h at 37 °C.

### 4.7. Cell Culture

Pneumocyte cell line A549, type II, obtained from a human lung carcinoma (LGC Standards, UK) was cultured in DMEM medium supplemented with 10% heat-inactivated fetal bovine serum (FBS) and 1% Antibiotic Antimycotic Solution (Sigma Aldrich). The culture was maintained at 37 °C in a humidified incubator with 5% CO_2_. Every three to four days, A549 cells were routinely passaged. Before infection with laboratory and carbapenem-resistant *A. baumannii* (S02, S04, S06), A549 cells were cultured in DMEM devoid of FBS and antibiotics after being thoroughly cleaned three times in preheated phosphate-buffered saline (PBS).

### 4.8. Assessment of Ceragenin Cytotoxicity towards A549 Epithelial Cells Using MTT Assay

To evaluate the cytotoxic effects of the tested ceragenins (CSA-13, CSA-44, and CSA-131) on A549 epithelial cells, a methyl thiazolyl tetrazolium (MTT) assay was conducted. In detail, A549 cells were seeded onto 48-well plates at a density of 15–20 × 10^3^ cells per well. Subsequently, the cells were treated with varying concentrations of ceragenins for 24 h. Following the treatment period, the cells were gently washed with phosphate-buffered saline (PBS) to remove any residual ceragenins. Following the washing step, the viability of the treated cells was assessed using the MTT assay. A solution of MTT to reach a final concentration of 0.5 mg/mL was added to each well, and the plates were then incubated for an additional 4 h at standard cell culture conditions. After the completion of the incubation period, the formazan crystals formed by metabolically active cells were solubilized using an appropriate solvent. Subsequently, the spectrophotometric absorbance of the solubilized formazan was measured at 550 nm using a Varioskan Lux microplate reader. The absorbance values obtained were then used to quantify the relative cell viability compared to samples without ceragenins. All experiments were performed in triplicate to ensure the reliability of the results. Statistical analysis was conducted to assess the significance of any observed differences between treatment groups.

### 4.9. Adhesion Assay

To assess the adherence of bacteria to the lung epithelial cells, A549 cells were infected with 10^8^ CFU/mL of laboratory (ATCC19606) and carbapenem-resistant *A. baumannii* (S02, S04, S06) for two hours at 37 °C in 5% CO_2_. After that, five washings with preheated PBS were performed on infected A549 cells, and 0.5% Triton X-100 was used to lyse them. The number of bacteria that attached to A549 cells was counted after diluted lysates were plated onto blood agar (Blood-Agar Columbia, Becton Dickinson Microbiology Systems, East Rutherford, NJ, USA) and incubated at 37 °C for 24 h.

### 4.10. Statistical Analysis

Collected data and differences were determined using the one-tailed Student’s *t*-test. *p* < 0.05 was considered to be statistically significant. The results are the average of three measurements.

## 5. Conclusions

Ceragenins, by efficiently destroying bacterial membranes and preventing biofilm development, show great promise as therapeutic agents for treating *A. baumannii* infections, especially against carbapenem-resistant strains. Important new information on the biophysical effects of ceragenins on bacterial cells was obtained in this work by the use of atomic force microscopy, and adhesion studies demonstrated their capacity to stop bacterial attachment to host cells. These results highlight the value of adhesion and AFM research in our knowledge of and progress toward ceragenins as new antibacterial therapies.

## Data Availability

The original contributions presented in the study are included in the article; further inquiries can be directed to the corresponding author/s.
